# Availability of Software-Based Correction of Mandibular Plane for the Vertical Measurement of the Mandible in Cone Beam Computed Tomography

**DOI:** 10.1155/2015/808625

**Published:** 2015-10-22

**Authors:** Sang-Sun Han, Kwang-Min Lee, Kee-Deog Kim

**Affiliations:** ^1^Department of Oral and Maxillofacial Radiology, College of Dentistry, Yonsei University, Seoul 03722, Republic of Korea; ^2^Department of Advanced General Dentistry, College of Dentistry, Yonsei University, Seoul 03722, Republic of Korea

## Abstract

*Objectives*. To investigate the availability of correction of mandibular plane using software for vertical measurements in cone beam computed tomography (CBCT) according to the sites of the mandible. *Methods*. CBCT scans of six dry mandibles were performed at 0-, 5-, 10-, 15-, and 20-degree angles relative to CBCT scanning table. Using the imaging software, mandibular planes of the different angles were corrected to that of 0-degree angle on the CBCT images. Before and after correction of the mandibular planes, the distance from the mandibular canal to the alveolar crest was measured at M1, M2, and M3 areas of the mandible and vertical measurements were statistically compared with those of 0-angle location using the paired *t*-test. *Results*. Prior to correction, the vertical measurements increased as the angle increased. The greatest differences of measurements were observed in M3 areas (*P* < 0.05). After correction, a strong correlation was found in measurements between the 0-degree angle and the other angles in all sites of the mandible (*P* > 0.05). *Conclusions*. The vertical measurements of CBCT were significantly influenced by mandibular positioning. When CBCT scans are performed at angles other than 0-degree angle, software-based correction of the mandibular plane can be a reliable tool for the accurate vertical measurements in CBCT.

## 1. Introduction

The assessment of the available bone height is one of the significant factors which influences the decision regarding the length of the implant prior to dental implant placement [1]. Computed tomography (CT) is an accurate imaging modality for the evaluation of preimplant sites in the mandibles [2]. Cone beam computed tomography (CBCT) also enables measurement of the distance between the alveolar crest and the mandibular canal so that impingement of the inferior alveolar nerve can be avoided [3–6].

Recently, due to the advantages of low radiation exposure and relatively low cost, CBCT scans have come to be preferred over CT for evaluations of bone quantity prior to dental implant placement [4, 7, 8]. Additionally, CBCT is known to provide measurements with submillimeter accuracy [9].

Our previous study reported that vertical measurements based on CT scans can be significantly influenced by mandibular positioning angle [10]. In CBCT, the accuracy of the measurements is affected by the CBCT system and software, patient motion during the scan, and the clinician's skill in interpreting the images [3]. However, we were unable to identify any study that focused on the influence of changes in the mandibular position on the vertical measurements from CBCT scans.

An imaging software program has been developed to improve the applicability of CBCT imaging for dental treatments [8, 11, 12]. Today, this software program has the functionality to make the adjustment of the axes of CBCT data that are obtained at angles other than the 0-degree angle. We thought that such functionality would make it possible to obtain the accurate vertical measurements regardless of the mandibular positions. However, no study has addressed the correction of mandibular plane using the software.

This study aimed at evaluating the influence of mandibular position changes on the vertical measurements from CBCT scans and investigating the availability of software-based correction of mandibular plane on CBCT images for vertical measurements according to the sites of the mandible.

## 2. Materials and Methods

### 2.1. CBCT Scans

**Figure 1:**
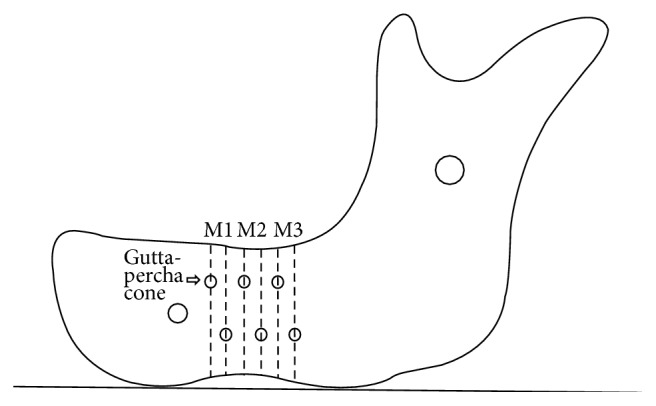
Marked sites of the mandible for the measurements.

The CBCT scans were conducted with reference to the experimental procedure that we have previously reported for CT scans [10]. Six dry mandibles in partially edentulous states were used. To evaluate the measurement differences according to sites of the mandibles, gutta-percha cones (1 × 1 mm) were attached as references to the areas at points M1 (5 and 10 mm distal to the mental foramen), M2 (15 and 20 mm distal to the mental foramen), and M3 (25 and 30 mm distal to the mental foramen) on the right and left buccal surfaces of the mandibles (Figure 1). To ensure the reproducibility of the CBCT scans with regard to different angles, the inferior border of the mandible was set on a 30 mm thick styrofoam plate that was fixed to an acrylic plate. To evaluate the influence of mandibular positional changes on the vertical measurements, the CBCT scans were performed in the positions described below.

The inferior border of the mandible (mandibular plane) was positioned parallel to the CBCT scanning table of 0-degree location and at the following positions:

1. At a positive 5-degree angle to the scanning table (5-degree location).
2. At a positive 10-degree angle to the scanning table (10-degree location).
3. At a positive 15-degree angle to the scanning table (15-degree location).
4. At a positive 20-degree angle to the scanning table (20-degree location).

An Alphard 3030 CBCT unit (ASAHI Co., Tokyo, Japan) was used. All images were recorded at 80 kVp and 5 mA over 17 s using a 102 × 102 mm field of view and an axial slice thickness of 0.2 mm. To obtain accurate results, the images of the remnant teeth and the extraction sockets were excluded.

### 2.2. Vertical Measurements of the CBCT Images before Correction of the Mandibular Plane

Before the correction of the mandibular plane, a total of 56 areas (18 M1 areas, 20 M2 areas, and 18 M3 areas) were obtained, and the 280 cross-sectional images taken from the 56 areas at 0-, 5-, 10-, 15-, and 20-degree angles were used.

**Figure 2:**
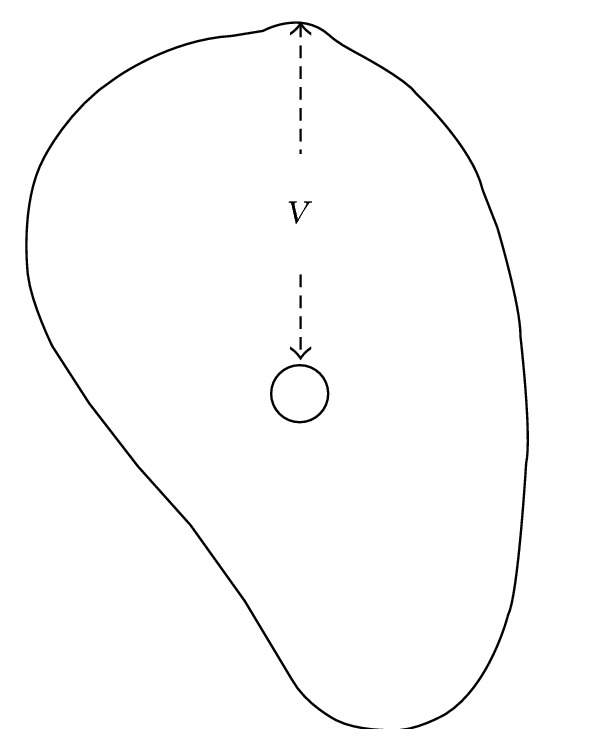
Measurement of the distance (*V*) from the top of the mandibular canal to the alveolar crest on cross-sectional image.

Using the In2Guide software OnDemand3D (Cybermed Inc., Seoul, Korea), the distances from the top of the mandibular canal to the alveolar crest were measured on the cross-sectional images at the marked areas at which the gutta-percha was highly visible (M1, M2, and M3) at the 0-, 5-, 10-, 15-, and 20-degree angles (Figure 2). All measurements from CBCT images were performed twice with an interval of three weeks by a single experienced oral and maxillofacial radiologist and the means of these measurements were adopted for analysis.

### 2.3. Vertical Measurements of the CBCT Images after Correction of the Mandibular Plane

**Figure 3:**
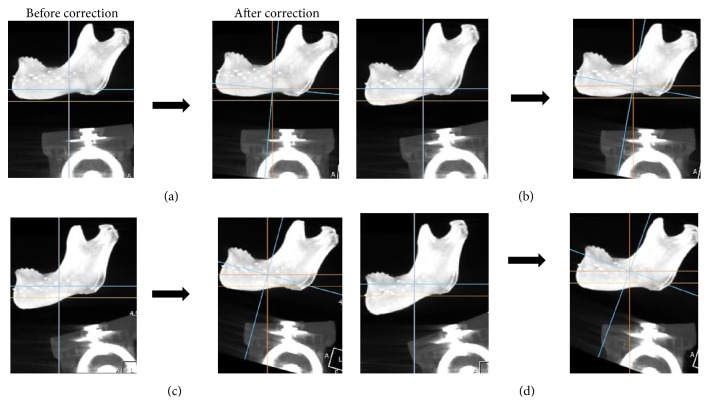
CBCT images before and after the correction of the mandibular planes. The inferior border of the mandible (mandibular plane) was positioned at 5-, 10-, 15-, and 20-degree angles relative to CBCT scanning table, and the mandibular planes at different angles were corrected to the 0-degree position using the software. (a) Five-degree angle location. (b) Ten-degree angle location. (c) Fifteen-degree angle location. (d) Twenty-degree angle location.

The imaging software OnDemand3D has the functionality to make the adjustment of the axes of CBCT data. This function is used for correction of the mandibular planes of CBCT images that are obtained at angles other than the 0-degree angle. In the adjustment of CBCT data, the base plane is dragged to reslice as newly aligned DICOM data. Rotation degrees will be shown on the 3D plane automatically. The mandibular planes in the CBCT images taken at the 5-, 10-, 15-, and 20-degree angles were corrected to that of the 0-degree position for M1, M2, and M3 areas of the mandibles using the software program (Figure 3) and 224 cross-sectional images were added. The vertical measurements were performed using the same method that was applied to the CBCT images prior to correction.

### 2.4. Statistical Analyses

To assess intraobserver difference, Wilcoxon matched-pairs test was used for repeated measurements of the same observer. All vertical measurements before and after correction were statistically compared with those obtained at 0-degree location according to the M1, M2, and M3 areas using the paired *t*-test (*P* < 0.05). The data set was analyzed using the Statistical Package for Social Science software ver. 19.0 (SPSS, Chicago, IL).

## 3. Results

There was no statistically significant intraobserver difference in repeated measurements of CBCT images (*P* > 0.05). Intraobserver consistency was rated at 95% between two measurements.

Before the correction of the mandibular plane, the value of vertical measurements increased as the angle between the mandibular plane and the scanning table increased (Table 1). The vertical measurements between the 0-degree and 5-degree angles were not statistically significant different in any site of the mandible (*P* > 0.05). However, at the 15-degree and 20-degree angles, there were statistically significant differences for the M2 and M3 areas (*P* < 0.05; Table 3).

The differences of vertical measurements were more pronounced in the M3 areas than in the M2 areas and the differences in M2 areas were greater than those in M1 areas (Table 3). In the M3 areas, statistically significant differences at 10-, 15-, and 20-degree angles were observed (*P* < 0.05). However, in the M1 area, there were no statistically significant differences between 0-degree angle and the other angles (*P* > 0.05; Table 3).

In contrast, after the correction of the mandibular plane with software, the vertical measurements were relatively constant across different angles of the mandibular planes regardless of sites of the mandible (Table 2). There was no statistically significant difference in the measurements between the 0-degree angle and the other angles (*P* > 0.05). Regarding the marked areas of the mandible, there was no statistically significant effect in the M1, M2, and M3 areas (*P* > 0.05; Table 4).

## 4. Discussion

During evaluations of preimplant sites of the mandible, accurate measurements of the distance from the mandibular canal to the alveolar crest on radiographs have been linked to primary implant success [13]. Insertion of an inadequately long implant can injure the inferior alveolar nerve resulting in permanent hypoesthesia of the lower lip [14].

Prior to the correction of mandibular planes, the vertical measurements based on CBCT scans were affected by changes in the position of the mandible. As the angle between the mandibular plane and the CBCT scanning table increased, the vertical measurements increased. Because the cross sections of CBCT images that were taken at angles other than the 0-degree angle were not perpendicular to the long axis of the mandible, the vertical measurements from the CBCT images taken at different angles might be overestimated compared to those at 0-degree angle location of the mandible.

Regarding the sites of the mandible, before correction, the greatest differences of vertical measurements were observed in the posterior regions, and the difference of measurements decreased toward the more anterior regions. Because the M3 areas are the farthest from the axis of rotation in terms of angle, the cross-sectional images of the areas were oblique to the long axis of the mandible. So the measurements in the M3 areas were likely greater than those of the other sites in the mandibles.

Large errors in measurements of available bone height might cause nerve injury during the insertion of the implant. When patients are not accurately positioned in CBCT scans, CBCT rescans might be necessary for accurate evaluations of preimplant site. When patients are not accurately positioned in CBCT scans, CBCT rescan might be necessary for accurate evaluation of preimplant site, and it results in unnecessary radiation exposure to the patient.

Today, imaging software has been developed for dental treatment, and the software has the functionality to rotate the axis of CBCT image. We thought that this function should be used for the correction of mandibular plane on all CBCT images that were at angles other than the 0-degree angle. After correction, the vertical measurements at different angles corresponded relatively well with those at the 0-degree angle. Additionally, a strong correlation was found in the vertical measurements between the 0-degree angle and the other angles in all sites of the mandible. Therefore, the correction of the mandibular plane using software is thought to be a reliable tool for the accurate measurements of the vertical distance in preimplant site of CBCT images.

In conclusion, changes of mandibular position in CBCT scan affected the vertical measurements from the cross-sectional images according to the sites of the mandible. However, when CBCT scans are performed at angles other than the 0-degree angle, software-based correction of the mandibular plane can provide satisfactory information about the vertical measurements without requiring an additional CBCT scanning.

## Figures and Tables

**Table 1:** Means and standard deviations of vertical measurements according to mandibular angles before correction (mm).

	0°	5°	10°	15°	20°
M1 (*n* = 18)	11.79 ± 1.44	11.84 ± 1.43	11.98 ± 1.38	12.12 ± 1.36	12.27 ± 1.46
M2 (*n* = 20)	10.12 ± 1.30	10.23 ± 1.30	10.56 ± 1.49	11.04 ± 1.54	11.34 ± 1.75
M3 (*n* = 18)	9.22 ± 1.64	9.45 ± 1.64	9.93 ± 1.87	10.52 ± 1.95	11.00 ± 2.12

**Table 2:** Means and standard deviations of vertical measurements according to mandibular angles after correction (mm).

	0°	5°	10°	15°	20°
M1 (*n* = 18)	11.79 ± 1.44	11.77 ± 1.51	11.78 ± 1.53	11.83 ± 1.46	11.76 ± 1.41
M2 (*n* = 20)	10.12 ± 1.30	10.21 ± 1.28	10.17 ± 1.32	10.18 ± 1.28	10.21 ± 1.28
M3 (*n* = 18)	9.22 ± 1.64	9.18 ± 1.74	9.19 ± 1.78	9.25 ± 1.71	9.25 ± 1.67

**Table 3:** Mean error and standard deviation between measurements at 0° location and others before correction (mm).

	5°	10°	15°	20°
M1 (*n* = 18)	0.04 ± 0.24	0.18 ± 0.48	0.29 ± 0.96	0.33 ± 0.58
M2 (*n* = 20)	0.10 ± 0.26	0.21 ± 0.55	0.91 ± 0.45^*∗*^	1.21 ± 0.69^*∗*^
M3 (*n* = 18)	0.19 ± 1.05	0.71 ± 0.54^*∗*^	1.31 ± 0.71^*∗*^	1.79 ± 0.94^*∗*^

^*∗*^Statistically significant difference at *P* < 0.05.

**Table 4:** Mean error and standard deviation between measurements at 0° location and others after correction (mm).

	5°	10°	15°	20°
M1 (*n* = 18)	0.02 ± 0.21	0.02 ± 0.29	0.04 ± 0.20	0.04 ± 0.25
M2 (*n* = 20)	0.09 ± 0.20	0.04 ± 0.18	0.06 ± 0.20	0.09 ± 0.24
M3 (*n* = 18)	0.04 ± 0.32	0.03 ± 0.32	0.03 ± 0.27	0.03 ± 0.23
